# Three-Dimensional Sectional Measurements of Shoulder Muscle Volume and Computed Tomography Density to Monitor Serial Postoperative Volume Changes in the Transverse Force Couple of Shoulder Muscles in Anterior Shoulder Instability

**DOI:** 10.7759/cureus.74963

**Published:** 2024-12-02

**Authors:** Keita Nagawa, Yuki Hara, Shinji Kakemoto, Taira Shiratori, Akane Kaizu, Masahiro Koyama, Kaiji Inoue, Katsunobu Sakaguchi, Eito Kozawa

**Affiliations:** 1 Radiology, Saitama Medical University, Saitama, JPN; 2 Orthopedics, Saitama Medical University, Saitama, JPN

**Keywords:** anterior shoulder instability, bankart repair, computed tomography density, muscle volume, sectional measurement

## Abstract

Purpose: In this study, we evaluated serial changes in shoulder muscle volume and computed tomography (CT) density of the transverse force couple (i.e., subscapularis (Ssc) vs. infraspinatus and teres minor (Isp+TM) muscles) after Bankart repair surgery in patients with anterior shoulder instability (ASI).

Methods: Four consecutive CT scans (obtained preoperatively and postoperatively at 1 month, 6 months, and 12 months) of 24 shoulders from 24 patients who underwent arthroscopic rotator cuff repair were examined. To generate muscle models, the Ssc and Isp+TM muscles were segmented. The reconstructed models were separated by the Y-view plane, and the planes were situated 2.5 cm and 5 cm medial to the Y-view plane. Balance was evaluated using volume ratio (VR_Ssc/Isp+TM_) and computed tomography-density ratio (CT-DR_Ssc/Isp+TM_) in each section of both groups, employing a three-dimensional sectional approach. Changes in these values were correlated with the preoperative background factors.

Results: Shoulder muscle volume and CT density were decreased in the early postoperative period compared with the preoperative period but recovered through the late postoperative period. The mean VR_Ssc/Isp+TM_ decreased in all sections in the early postoperative period but increased thereafter, whereas the mean CT-DR_Ssc/Isp+TM_ in all sections remained mostly unchanged throughout the perioperative period. Furthermore, the perioperative differences in muscle volume were correlated with sex, work and sports activity level, Hill-Sachs lesion, and range of motion. Perioperative differences in CT density were not correlated with most items, except for sulcus signs.

Conclusion: Serial changes in the VR_Ssc/Isp+TM_ group were significantly different and had clinical implications, whereas changes in the CT-DR_Ssc/Isp+TM_ group were not significantly different. This method may provide a potential indicator for evaluating muscle balance and recovery after Bankart repair surgery for ASI.

## Introduction

Anterior shoulder instability (ASI) is a pathological condition in which soft tissue or bone injury of the shoulder causes the humeral head to subluxate or dislocate from the glenoid fossa. Young, active, and athletic individuals are at risk for ASI, with a lifetime risk of 1%-2% [1,2]. Bankart injuries and Hill-Sachs deformities are well-known lesions associated with ASI. The treatment of ASI has evolved due to advances in arthroscopic techniques and an improved understanding of shoulder anatomy and its complex biomechanics [3,4].

Several important factors predict the surgical and clinical outcomes of shoulder disease. The degree of muscle atrophy and fatty degeneration are prognostic factors that determine postoperative structural and functional outcomes [5,6]. Many studies have focused on rotator cuff repair after shoulder rotator cuff tears, and improvements in muscle atrophy and fatty infiltration after surgery have been reported by several researchers [7-9]. These studies have demonstrated the utility of specific parameters, such as the cross-sectional area and occupancy of the shoulder muscles in oblique sagittal images (called the Y-view), tangent sign, and Goutallier classification [7-9]. These methods are mostly performed on a single image slice in oblique sagittal magnetic resonance imaging using the conventional Y-view plane. Two-dimensional (2D) evaluation approaches may cause interpretation errors for several reasons, including difficulty in selecting identical image slices and the migration of rotator cuff muscle volume after surgery [10,11].

Three-dimensional (3D) volumetric measurements of the shoulder muscles have been reported and may address the limitations of 2D assessments. Chung et al. [12] proposed 3D sectional volume measurement of the supraspinatus muscle along the Y-view plane and at 1 cm and 2 cm medial to the Y-view plane to evaluate serial muscle volume changes after arthroscopic rotator cuff repair. They reported an increase in supraspinatus muscle volume after surgery, with a larger increase observed in cases of large rotator cuff tears and successful healing [12]. Three-dimensional sectional volume measurements of the shoulder muscles may therefore be useful for accurately evaluating perioperative muscle volume changes.

Some studies have investigated shoulder muscle atrophy and fatty degeneration [13-15]. Ishikawa et al. [14] evaluated shoulder muscle cross-sectional areas in patients with anterior, posterior, and multidirectional shoulder instabilities. They found that the infraspinatus + teres minor (Isp+TM) muscle area was smaller than the subscapularis (Ssc) muscle area in anterior instability, whereas the Ssc muscle area was smaller than the Isp+TM muscle area in posterior and multidirectional instabilities [14]. An imbalance in the transverse force couple of the shoulder muscles (i.e., Ssc vs. Isp+TM) is an important factor in the interpretation of shoulder joint instability. In previous studies, the volume ratio between Ssc and Isp+TM (VR_Ssc/Isp+TM_) was 1.02 in healthy participants, regardless of age or sex [16]. Therefore, evaluating the muscular imbalance of the transverse force couple using the VR_Ssc/Isp+TM_ is meaningful because this ratio may be related to the muscular force balance and the direction of shoulder instability.

Our group previously examined VR_Ssc/Isp+TM_ using sectional volume measurements [15]. The VR_Ssc/Isp+TM_ was 1.13 in shoulders with ASI, which was higher than in nonpathological shoulders [15]. The computed tomography (CT)-density ratio between Ssc and Isp+TM (CT-DR_Ssc/Isp+TM_) was also investigated. CT-DR_Ssc/Isp+TM_ was 1.06 and 0.92 in the nonpathological shoulder and in the shoulder with ASI, respectively [15]. These ratios may reflect imbalances in volume and muscle properties, such as degeneration and edema. Although these muscular imbalances can be used to estimate the direction and extent of shoulder instability, most previous studies have examined the preoperative muscular imbalance of the transverse force couple [13-15]. Considering the relationship between muscle imbalance and shoulder instability, examining changes in muscle imbalance pre- and postoperatively is valuable. However, research on the perioperative status of muscular imbalances is limited.

Therefore, the purpose of this study was to examine serial changes in VR_Ssc/Isp+TM_ and CT-DR_Ssc/Isp+TM_ in patients with ASI during the perioperative period of Bankart repair surgery. Additionally, we aimed to identify the factors associated with these ratio changes.

## Materials and methods

Participants

This study was approved by the Research Ethics Committee of Saitama Medical University Hospital (Saitama, Japan; approval number 2022-072). All experiments were conducted in accordance with the relevant guidelines and regulations. The requirement for informed consent was waived due to the retrospective nature of the study.

**Figure 1:**
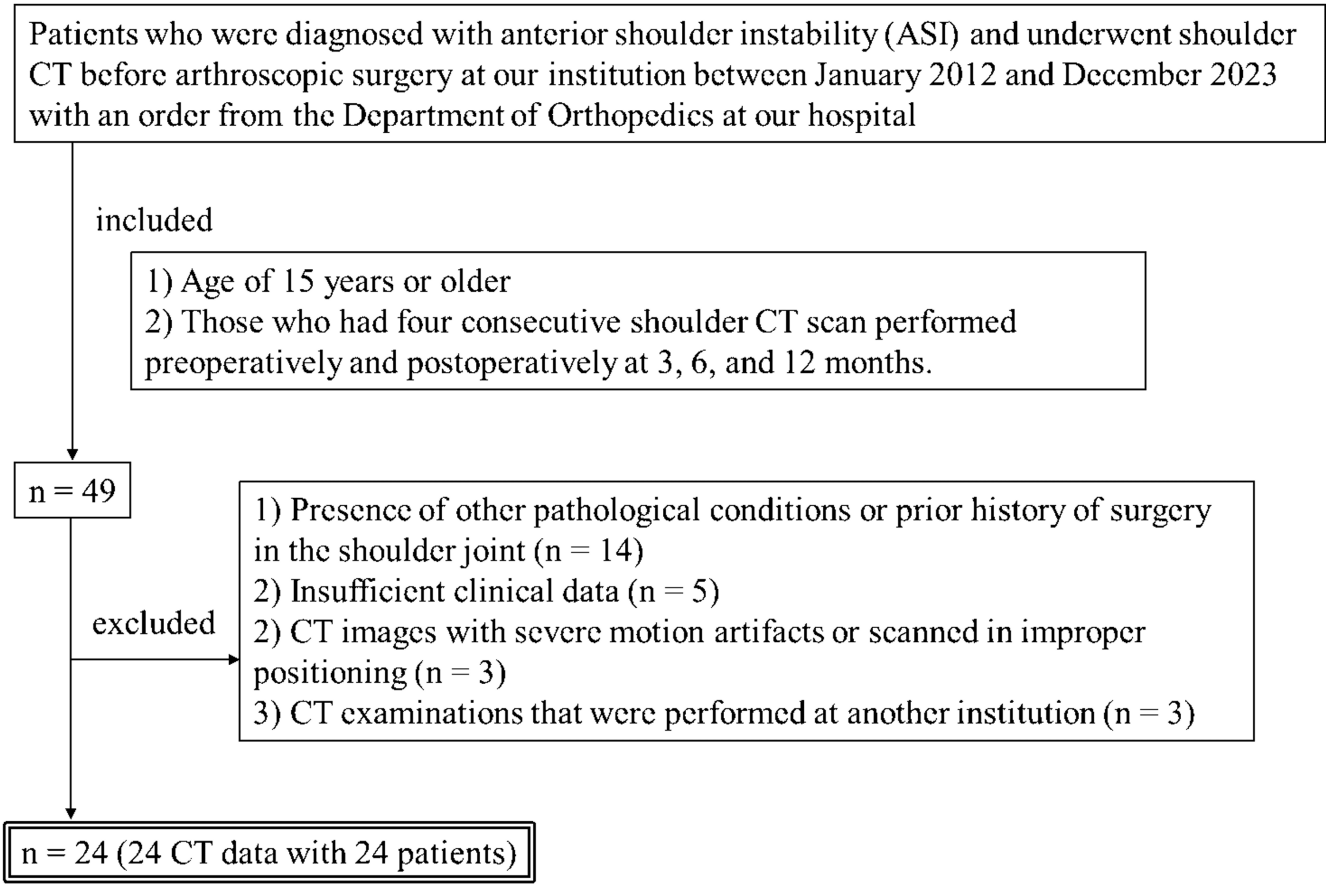
Flow chart of the inclusion and exclusion criteria for the study. CT: computed tomography.

Patients diagnosed with ASI who underwent shoulder CT before arthroscopic surgery at our institution between January 2012 and December 2023, with an order from the Department of Orthopedics at our hospital, were identified and reviewed. Figure 1 presents the inclusion and exclusion criteria. A total of 24 CT images from 24 patients were analyzed.

Demographic data

Work and sports activity levels were defined as “high” (i.e., physical labor or high-activity sports) or “low” (i.e., sedentary job and no sports activity). The load and shift tests were assessed as follows: grade 0 (i.e., mild translation), grade 1 (i.e., the humeral head rides up to the glenoid rim), grade 2 (i.e., the humeral head overrides the rim but spontaneously reduces), and grade 3 (i.e., the humeral head overrides the rim and remains dislocated). The sulcus sign was graded based on the degree of inferior translation: grade 0 (i.e., minimal laxity), grade 1 (i.e., <1 cm), grade 2 (i.e., 1-2 cm), and grade 3 (i.e., >2 cm). Orthopedic surgeons at our facility evaluated the load and shift tests and sulcus sign. Hill-Sachs lesions were classified as “none,” “shallow” (i.e., depression depth <5 mm), or “deep” (i.e., depression depth ≥5 mm). Bankart lesions were classified as “soft tissue type” (i.e., no significant changes in the bone) or “bone type” (i.e., bone defects or bone fragments). Hill-Sachs lesions and Bankart lesions were evaluated on preoperative CT and magnetic resonance imaging scans by two radiologists (Y.H. and K.N.) with 10 years and 9 years of experience, respectively.

CT acquisition

CT was conducted using the 128-multislice SOMATOM Definition Flash scanner (Siemens Healthcare, Erlangen, Germany). The patients were examined in the supine position with their arms adducted and lying on their abdomen. CT was conducted using the standard dedicated CT protocol for shoulders at our institution. The slice thickness was 2 mm, with reformations in the axial, coronal, and sagittal planes. The field of view was 15 cm × 15 cm × 9 cm.

Measurement of the volume and CT density of the shoulder muscle

**Figure 2:**
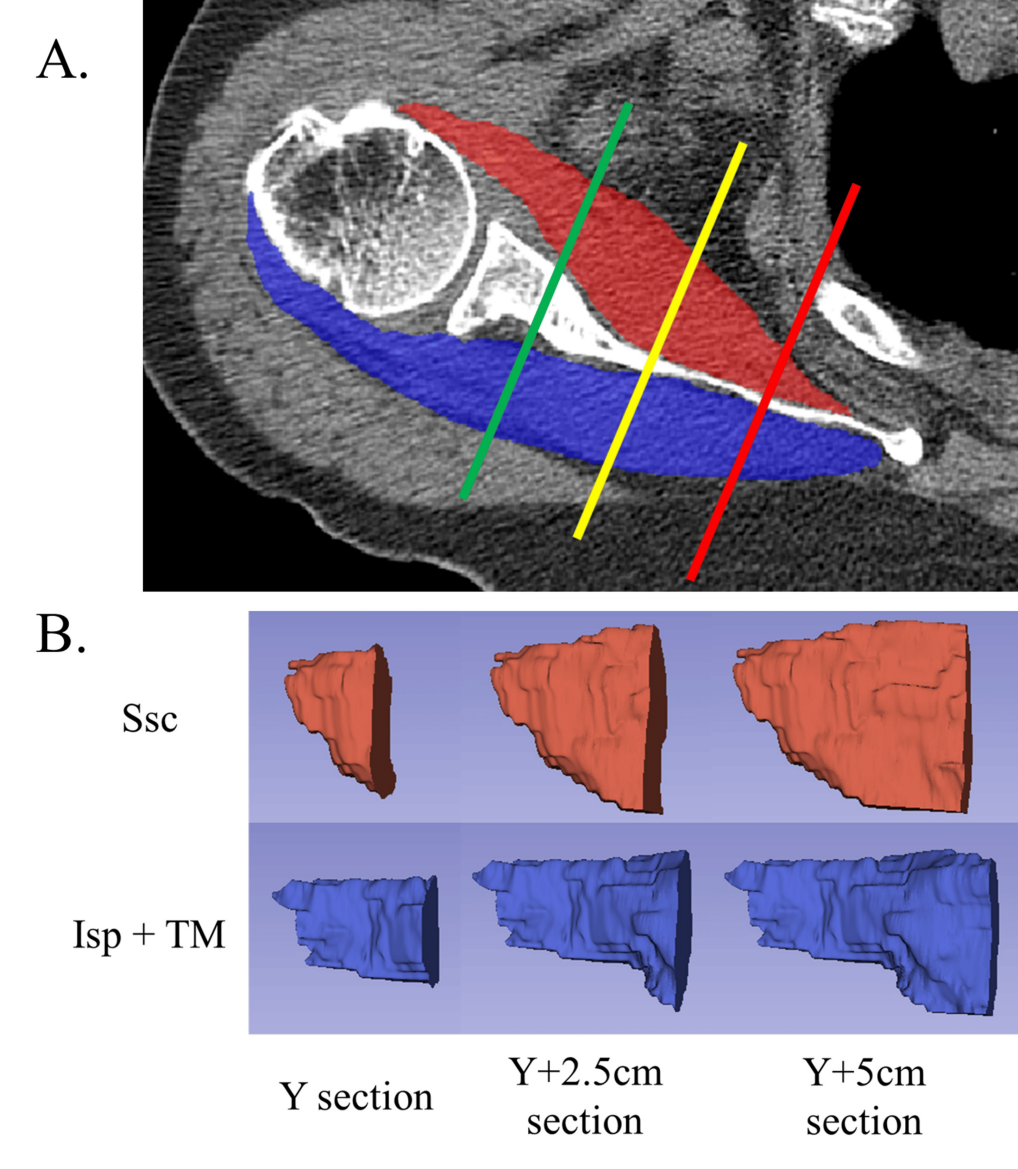
Measurement of the volume and CT density of the shoulder muscle. (A) The areas of the subscapularis muscle (Ssc, red area) and infraspinatus and teres minor muscles (Isp+TM, blue area) in every axial image are delineated using semi-automatic segmentation tools. The three-dimensional (3D) models of the Ssc and Isp+TM are separated by the Y-view (Y) plane (green line), the Y+2.5 cm plane (yellow line), and the Y+5 cm plane (red line). Additionally, a cut-off was implemented at the plane 3 cm inferior to the lower end of the glenoid labrum (not shown in the figure) to exclude the inferior margin of each muscle volume, as this area is usually out of range in our shoulder CT protocol. (B) For each 3D model of the Ssc and Isp+TM muscles, three sections of 3D models were newly created (resulting in a total of six sectional muscle models): from the most lateral end of the muscles to the Y-view plane (i.e., Y section), from the plane 2.5 cm medial to the Y-view (i.e., Y+2.5 cm section), and from the plane 5 cm medial to the Y-view (i.e., Y+5 cm section). These images were created by the authors for this manuscript. CT, computed tomography.

Shoulder muscle segmentation was performed using open-source software (ITK-SNAP version 3.8.0; University of Pennsylvania, Philadelphia, Pennsylvania). After the CT scans were loaded into ITK-SNAP in the Digital Imaging and Communications in Medicine format, the Ssc and Isp+TM areas were delineated on every transverse slice using semiautomatic segmentation tools (Figure 2). From the delineated contours, a 3D model of each shoulder muscle was reconstructed and saved as a standard 3D model in the Neuroimaging Informatics Technology Initiative (NIFTI) file format (*.nii.gz).

The 3D muscle sectioning was conducted as described in our previous study [15]. Cross-sectional muscle cut-offs were implemented at the following two specific planes: from the most lateral end of the muscles to the plane of the Y-view (i.e., Y section) and to the plane 5 cm medial to the Y-view (i.e., Y+5 cm section). Additionally, a cut-off at the plane 3 cm inferior to the lower end of the glenoid labrum was used to exclude the inferior margin of each muscle volume, as this area was typically out of range in our shoulder CT protocol. By implementing these adjustments, sectional muscle models were created, and their volumes and CT densities were determined. Ratios of muscle volume to CT density between the Ssc and Isp+TM (i.e., VR_Ssc/Isp+TM_ and CT-DR_Ssc/Isp+TM_) were then calculated for each section. All procedures were conducted using open-source software (3D Slicer 5.0.3; Kitware, Inc., Clifton Park, New York).

To assess interobserver reproducibility in the segmentation and measurement processes, two radiologists (Y.H. and K.N.), with 10 and 9 years of experience, respectively, independently performed these steps. Both radiologists were blinded to the clinical information. The intraclass correlation coefficient was measured to evaluate interobserver reproducibility.

Evaluation and statistical analysis

Serial changes in shoulder muscle volume and CT density before and after arthroscopic Bankart repair were examined at four different timepoints. The paired t-test was used to compare changes in the volume and CT density of the muscles preoperatively and at 1 month, 6 months, and 12 months postoperatively.

We examined the differences in muscle volumes between two timepoints, particularly between preoperative and 1 month postoperative (Dif.pre.1mo), 1 month and 12 months postoperatively (Dif.1.12mo), and preoperative and 12 months postoperative (Dif.pre.12mo). Correlations were evaluated using the Pearson correlation test.

Additionally, we analyzed the correlation between perioperative differences (i.e., Dif.pre.1mo, Dif.1.12mo, and Dif.pre.12mo) in muscle volume and CT density for the Ssc and Isp+TM groups and clinical and demographic parameters (e.g., age, sex, symptom duration, work, and sports activity level) using multivariate analysis.

Statistical analyses were conducted using the open-source software package Python (scikit-learn version 0.22.1, Rocquencourt, France). Statistical significance was set at P < 0.05.

## Results

This study included 24 CT scans from 24 patients. Demographic and clinical data are summarized in Table 1. Postoperative CT evaluations were conducted at 1 month (40.2 ± 25.7 days), 6 months (195.1 ± 16.4 days), and 12 months (412.7 ± 48.9 days). Interobserver reproducibility was good for all 3D volumes, with mean intraclass correlation coefficient values of 0.838 preoperatively and 0.822, 0.846, and 0.813 at 1 month, 6 months, and 12 months postoperatively.

**Table 1:** Demographic and clinical characteristics of the study population Data are presented as the number (%) of patients, unless otherwise indicated.

Variables	Values
Age, years, mean ± SD	23.4 ± 8.7
Sex, male	19 (79)
Side, right	10 (42)
Symptom duration, months, mean ± SD	72.9 ± 92.2
Work and sports activity level, high	17 (71)
Load and shift test, grade, mean ± SD	1.8 ± 0.8
Sulcus sign, grade, mean ± SD	1.0 ± 0.8
Hill–Sachs lesion, none/shallow/deep	1/15/8
Bankart lesion, soft tissue/bone	4/20
Preoperative range of motion, degrees, mean ± SD	
Forward flexion	159.8 ± 26.8
Abduction	155.91 ± 27.9
External rotation	54.1 ± 16.4

Table 2 summarizes the serial changes in shoulder muscle volume and CT density during the perioperative period after arthroscopic Bankart repair. Overall, the mean 3D muscle volumes of the Ssc and Isp+TM decreased at 1 month postoperatively compared with their preoperative values, with statistical significance observed only in the Y+5 cm sections of the Ssc and Isp+TM. The mean CT density of Ssc and Isp+TM also decreased at 1 month postoperatively compared with the preoperative values in the Y and Y+5 cm sections, with statistical significance. No significant changes were observed, but a slight recovery in the volume and CT density of both muscles occurred between 1 month and 6 months postoperatively and between 1 month and 12 months postoperatively. The mean VR_Ssc/Isp+TM_ decreased in all sections at 1 month postoperatively compared with the preoperative values but increased afterward, although these changes were not statistically significant. The mean CT-DR_Ssc/Isp+TM_ in all sections remained mostly unchanged throughout the perioperative period.

**Table 2:** Serial changes in shoulder muscle volume and computed tomography (CT) density during the perioperative period of arthroscopic Bankart repair. Data are presented as the mean ± the standard deviation. Sectional measurements of muscle volume and CT density were conducted as follows: from the most lateral end of the muscles to the plane of the Y-view (i.e., Y section) and from the most lateral end of the tendons to the plane 5 cm medial to the Y-view (i.e., Y+5 cm section). The paired t-test was used to compare between the two groups. ^a^Comparison between the values obtained preoperatively and at 1 month postoperatively. ^b^Comparison between the values obtained at 1 month and 6 months postoperatively. ^c^Comparison between the values obtained at 1 month and 12 months postoperatively. HU, Hounsfield units.

Variables	Category	Preoperatively	1 Month after surgery		6 Months after surgery		12 Months after surgery	
		Value	Value	P-value^a^	Value	P-value^b^	Value	P-value^c^
Volume, cm^3^								
Subscapularis	Y section	33.5 ± 9.5	27.3 ± 8.0	0.051	34.8 ± 8.5	0.246	31.1 ± 8.6	0.480
	Y+5 cm section	109.2 ± 18.2	97.7 ± 17.4	<0.001	107.9 ± 19.8	0.477	101.5 ± 20.1	0.460
Infraspinatus + teres minor	Y section	33.9 ± 11.0	35.2 ± 9.6	0.865	35.4 ± 9.6	0.651	31.8 ± 8.9	0.806
	Y+5 cm section	96.0 ± 18.3	91.9 ± 19.0	0.034	97.3 ± 16.1	0.846	91.8 ± 19.2	0.634
CT density, HU								
Subscapularis	Y section	59.2 ± 6.1	54.7 ± 5.0	0.002	60.3 ± 6.9	0.707	59.1 ± 4.9	0.404
	Y+5 cm section	59.1 ± 4.5	56.7 ± 4.6	0.029	60.3 ± 4.7	0.304	58.4 ± 3.6	0.456
Infraspinatus + teres minor	Y section	59.0 ± 6.1	56.2 ± 7.1	0.043	58.6 ± 6.1	0.921	58.7 ± 5.6	0.438
	Y+5 cm section	64.8 ± 5.1	62.4 ± 5.9	0.022	64.6 ± 5.3	0.762	64.1 ± 5.1	0.467
The ratio of volume between subscapularis and infraspinatus + teres minor								
	Y section	1.13 ± 0.53	0.81 ± 0.24	0.301	1.04 ± 0.35	0.249	1.04 ± 0.38	0.431
	Y+5 cm section	1.16 ± 0.17	1.08 ± 0.15	0.103	1.11 ± 0.16	0.178	1.12 ± 0.18	0.157
The ratio of CT density between subscapularis and infraspinatus + teres minor								
	Y section	1.01 ± 0.09	0.98 ± 0.09	0.308	1.04 ± 0.12	0.954	1.01 ± 0.06	0.930
	Y+5 cm section	0.91 ± 0.04	0.91 ± 0.04	0.471	0.94 ± 0.06	0.529	0.91 ± 0.03	0.497

Table 3 summarizes the differences in muscle volume and CT density (i.e., Dif.pre.1mo, Dif.1.12mo, and Dif.pre.12mo) and the correlations between the two perioperative periods. A negative correlation was observed between Dif.pre.1mo and Dif.1.12mo for all sections and variables, with statistical significance, except for the Y section of Ssc. A positive correlation was observed between Dif.pre.1mo and Dif.pre.12mo for all variables, although some correlations were not statistically significant.

**Table 3:** Comparison between perioperative muscle volume differences of the infraspinatus + teres minor and clinical and demographic parameters using multivariate analysis Multivariate analysis was conducted to assess the correlation between the volume differences of the infraspinatus and teres minor muscles during the perioperative period (i.e., between preoperatively and 1 month after surgery (Dif.pre.1mo), between 1 and 12 months after surgery (Dif.1.12mo), and between preoperatively and 12 months after surgery (Dif.pre.12mo)) and clinical and demographic parameters. The sectional measurement was conducted as follows: from the most lateral end of the muscles to the plane of the Y-view (i.e., Y section) and from the most lateral end of the tendons to the plane 5 cm medial to the Y-view (i.e., Y+5 cm section). RC, regression coefficient.

Variables	Dif.pre.1mo				Dif.1.12mo				Dif.pre.12mo			
	Y section		Y+ 5 cm section		Y section		Y+ 5 cm section		Y section		Y+ 5 cm section	
	RC	P value	RC	P value	RC	P value	RC	P value	RC	P value	RC	P value
Age	-1238.36	0.017	-1720.20	0.075	-99.53	0.830	788.78	0.682	-1946.65	0.172	-4640.02	0.330
Sex	-2477.02	0.002	-2907.66	0.026	3486.27	0.120	2197.41	0.947	-1154.34	0.239	-4684.73	0.283
Side	4304.55	0.114	4633.76	0.386	-1293.78	0.205	-684.95	0.975	-1094.04	0.272	-4178.59	0.337
Symptom duration	92.03	0.026	108.15	0.150	19.90	0.557	-32.66	0.788	133.40	0.165	268.10	0.365
Work and sports activity level	-1245.94	0.004	-1595.55	0.028	2214.45	0.172	3292.95	0.915	-2723.70	0.414	5935.18	0.663
Bankart lesion	1166.00	0.053	7672.62	0.479	8563.51	0.141	3397.34	0.819	1981.63	0.179	5144.66	0.317
Hill–Sachs lesion	-1367.78	0.040	-1511.04	0.219	2475.68	0.181	-6481.04	0.859	-1207.34	0.206	-3775.02	0.308
Load and shift test	2072.78	0.328	5598.75	0.234	-1136.34	0.178	-635.58	0.969	-4013.88	0.364	-1632.25	0.421
Sulcus sign	-2998.40	0.140	-3532.49	0.384	3821.99	0.245	-1223.78	0.875	-5193.75	0.373	-3195.33	0.310
Range of motion												
Forward flexion	-412.01	0.015	-100.25	0.698	815.86	0.165	-896.20	0.486	-260.97	0.299	-1445.11	0.269
Abduction	794.32	0.005	478.14	0.234	-1476.16	0.169	1167.31	0.597	513.65	0.215	2029.39	0.262
External rotation	-471.51	0.061	-345.76	0.384	847.48	0.217	-878.06	0.597	-898.43	0.206	-3472.02	0.256

Finally, we evaluated the relationship between perioperative differences in muscle volume and CT density for Ssc and Isp+TM and clinical and demographic parameters using multivariate analysis. The results are summarized in Tables 4-7.

**Table 4:** The differences of muscle volume and computed tomography (CT) density between the two perioperative periods and their correlations Data are presented as the mean ± the standard deviation. Differences in muscle volume and computed tomography (CT) density were evaluated between preoperatively and 1 month postoperatively (Dif.pre.1mo), between 1 month and 12 months postoperatively (Dif.1.12mo), and between preoperatively and 12 months postoperatively (Dif.pre.12mo). The sectional measurement was performed as follows: from the most lateral end of the muscles to the plane of the Y-view (i.e., Y section) and from the most lateral end of the tendons to the plane 5 cm medial to the Y-view (i.e., Y+5 cm section). The Pearson correlation test was used to compare between the two groups. ^a^Correlation between Dif.pre.1mo and Dif.1.12mo. ^b^Correlation between Dif.pre.1mo and Dif.pre.12mo. HU: Hounsfield units.

Variables	Category	Between preoperatively and 1 month after surgery	Between 1 and 12 months after surgery	Correlation		Between preoperatively and 12 months after surgery	Correlation	
				Value	P-value^a^		Value	P-value^b^
Volume, cm^3^								
Subscapularis	Y section	-5.89 ± 9.82	5.41 ± 7.71	-0.314	0.063	-0.22 ± 10.68	0.712	<0.001
	Y+5 cm section	-12.15 ± 11.17	9.73 ± 9.93	-0.571	0.009	-2.04 ± 8.03	0.401	0.080
Infraspinatus + teres minor	Y section	0.84 ± 8.56	-1.70 ± 5.48	-0.608	0.004	0.37 ± 5.37	0.537	0.015
	Y+5 cm section	-5.21 ± 11.06	4.47 ± 11.08	-0.712	<0.001	1.12 ± 6.74	0.348	0.132
CT density, HU								
Subscapularis	Y section	-4.55 ± 5.73	5.10 ± 4.12	-0.288	0.280	0.56 ± 4.80	0.784	<0.001
	Y+5 cm section	-2.51± 5.00	2.84 ± 4.07	-0.566	0.009	-0.01 ± 3.09	0.405	0.076
Infraspinatus + teres minor	Y section	-2.63 ± 6.59	2.89 ± 6.53	-0.695	0.003	-0.82 ± 4.64	0.279	0.234
	Y+5 cm section	-2.34 ± 5.61	2.61 ± 5.55	-0.492	0.028	-0.79 ± 4.50	0.241	0.305

**Table 5:** Comparison between perioperative muscle volume differences of the subscapularis and clinical and demographic parameters by using multivariate analysis Multivariate analysis was conducted to assess the correlation between the volume differences of the subscapularis muscle during the perioperative period (i.e., between preoperatively and 1 month after surgery (Dif.pre.1mo), between 1 and 12 months after surgery (Dif.1.12mo), and between preoperatively and 12 months after surgery (Dif.pre.12mo)) and clinical and demographic parameters. The sectional measurement was conducted as follows: from the most lateral end of the muscles to the plane of the Y-view (i.e., Y section) and from the most lateral end of the tendons to the plane 5 cm medial to the Y-view (i.e., Y+5 cm section). RC, regression coefficient.

Variables	Dif.pre.1mo				Dif.1.12mo				Dif.pre.12mo			
	Y section		Y+ 5 cm section		Y section		Y+ 5 cm section		Y section		Y+ 5 cm section	
	RC	P value	RC	P value	RC	P value	RC	P value	RC	P value	RC	P value
Age	1865.47	0.149	1413.79	0.142	-1201.89	0.582	-1013.65	0.757	293.24	0.874	-271.86	0.302
Sex	2268.68	0.871	3758.53	0.718	-9849.11	0.788	-1484.04	0.802	-3964.04	0.048	-2110.03	0.003
Side	-4561.58	0.540	6305.95	0.276	-1453.00	0.576	-3470.70	0.927	-6653.87	0.694	-3288.10	0.205
Symptom duration	-92.56	0.354	-82.67	0.274	-15.05	0.907	13.39	0.949	-25.88	0.831	-18.84	0.282
Work and sports activity level	8206.59	0.320	-2964.68	0.615	7452.79	0.824	-4256.26	0.937	-4416.85	0.701	-6120.37	0.037
Bankart lesion	-1379.00	0.379	-1677.04	0.175	3667.00	0.338	5940.40	0.125	-1270.12	0.394	-2128.85	0.113
Hill–Sachs lesion	5344.17	0.741	2161.74	0.857	3094.00	0.937	-1000.02	0.875	-1547.01	0.450	-2173.04	0.008
Load and shift test	2143.27	0.731	5690.37	0.251	-2432.86	0.891	2522.14	0.930	2870.43	0.700	2455.84	0.088
Sulcus sign	-1875.70	0.737	855.31	0.836	-1339.00	0.294	-7369.81	0.618	-1831.98	0.119	-1237.02	0.005
Range of motion												
Forward flexion	6.65	0.985	525.40	0.097	-407.23	0.736	-849.50	0.672	-1305.68	0.112	-605.36	0.009
Abduction	-203.68	0.702	-722.70	0.111	121.69	0.955	1190.65	0.743	1486.10	0.182	969.43	0.008
External rotation	293.39	0.648	979.86	0.081	-738.09	0.669	-1479.32	0.608	-1027.25	0.362	-1294.36	0.007

**Table 6:** Comparison between perioperative differences in computed tomography (CT) density of the subscapularis muscle and clinical and demographic parameters using multivariate analysis Multivariate analysis was conducted to assess the correlation between the CT density differences in the subscapularis muscle during the perioperative period (i.e., preoperatively and 1 month after surgery (Dif.pre.1mo), between 1 month and 12 months after surgery (Dif.1.12mo), and between preoperatively and 12 months after surgery (Dif.pre.12mo)) and clinical and demographic parameters. The sectional measurement was conducted as follows: from the most lateral end of the muscles to the plane of the Y-view (i.e., Y section) and from the most lateral end of the tendons to the plane 5 cm medial to the Y-view (i.e., Y+5 cm section). RC, regression coefficient.

Variables	Dif.pre.1mo				Dif.1.12mo				Dif.pre.12mo			
	Y section		Y+ 5 cm section		Y section		Y+ 5 cm section		Y section		Y+ 5 cm section	
	RC	P value	RC	P value	RC	P value	RC	P value	RC	P value	RC	P value
Age	-1.19	0.119	-0.84	0.143	-0.27	0.927	-1.20	0.122	-2.60	0.336	-2.22	0.345
Sex	-11.56	0.194	-11.14	0.116	-8.43	0.607	7.77	0.319	-17.36	0.405	-13.54	0.441
Side	-10.94	0.042	-8.53	0.039	-1.11	0.973	-12.46	0.139	-24.96	0.326	-19.82	0.354
Symptom duration	0.06	0.281	0.04	0.317	-0.01	0.980	0.07	0.145	0.16	0.363	0.14	0.359
Work and sports activity level	-3.55	0.449	-5.01	0.189	1.62	0.941	17.23	0.143	13.68	0.256	8.79	0.335
Bankart lesion	10.21	0.274	7.29	0.302	-1.12	0.967	-5.46	0.859	20.94	0.417	12.33	0.543
Hill–Sachs lesion	-9.43	0.338	-7.90	0.269	2.32	0.662	20.60	0.141	3.51	0.810	6.06	0.653
Load and shift test	-2.23	0.544	-0.17	0.951	3.01	0.888	-8.17	0.160	-10.83	0.377	-7.74	0.439
Sulcus sign	-6.91	0.074	-6.94	0.031	-1.75	0.927	-2.55	0.236	-15.32	0.359	-12.35	0.384
Range of motion												
Forward flexion	-0.19	0.395	-0.11	0.518	-0.53	0.375	0.03	0.881	-0.70	0.312	-0.47	0.385
Abduction	0.46	0.179	0.38	0.151	0.55	0.428	-0.66	0.236	0.59	0.450	0.29	0.631
External rotation	-0.83	0.066	-0.63	0.065	-0.53	0.652	0.22	0.459	-1.18	0.396	-0.71	0.515

**Table 7:** Comparison between perioperative differences in computed tomography (CT) density of the infraspinatus + teres minor muscles and clinical and demographic parameters by using multivariate analysis Multivariate analysis was conducted to assess the correlation between the CT density differences in the infraspinatus and teres minor muscles during the perioperative period (i.e., preoperatively and 1 month after surgery (Dif.pre.1mo), between 1 month and 12 months after surgery (Dif.1.12mo), and between preoperatively and 12 months after surgery (Dif.pre.12mo)) and clinical and demographic parameters. The sectional measurement was conducted as follows: from the most lateral end of the muscles to the plane of the Y-view (i.e., Y section) and from the most lateral end of the tendons to the plane 5 cm medial to the Y-view (i.e., Y+5 cm section). RC, regression coefficient.

Variables	Dif.pre.1mo				Dif.1.12mo				Dif.pre.12mo			
	Y section		Y+ 5 cm section		Y section		Y+ 5 cm section		Y section		Y+ 5 cm section	
	RC	P value	RC	P value	RC	P value	RC	P value	RC	P value	RC	P value
Age	-0.54	0.512	-0.34	0.567	-2.42	0.477	-2.01	0.346	-0.80	0.737	-1.55	0.576
Sex	-18.81	0.100	-9.37	0.227	12.50	0.813	3.89	0.890	-1.08	0.955	-8.29	0.706
Side	-9.00	0.125	-7.69	0.084	-19.92	0.592	-14.58	0.496	-6.46	0.766	-12.11	0.626
Symptom duration	0.02	0.795	0.01	0.943	0.16	0.484	0.14	0.321	0.05	0.746	0.11	0.547
Work and sports activity level	-5.11	0.374	-3.50	0.401	23.29	0.651	18.32	0.536	7.71	0.468	8.42	0.466
Bankart lesion	12.63	0.265	7.60	0.348	-53.05	0.577	-28.56	0.450	-7.99	0.749	0.43	0.987
Hill–Sachs lesion	-16.12	0.195	-10.33	0.246	38.38	0.550	30.90	0.423	21.88	0.355	18.71	0.426
Load and shift test	-1.40	0.750	-0.14	0.964	-13.42	0.628	-11.17	0.493	-1.17	0.915	-5.04	0.688
Sulcus sign	-12.36	0.021	-9.56	0.017	-3.72	0.765	-3.40	0.630	-4.91	0.745	-9.60	0.585
Range of motion												
Forward flexion	-0.32	0.244	-0.06	0.736	0.26	0.878	0.09	0.131	-0.15	0.792	-0.20	0.753
Abduction	0.64	0.129	0.30	0.296	-1.25	0.704	-0.94	0.610	-0.30	0.709	-0.26	0.764
External rotation	-0.86	0.100	-0.62	0.101	0.62	0.795	0.65	0.633	0.42	0.751	0.24	0.864

With regard to the muscle volume difference of the subscapularis (Table 4), significant correlations were observed with sex, work and sports activity level, Hill-Sachs lesion, sulcus sign, and preoperative range of motion for Dif.pre.12mo in the Y+5 cm section.

With regard to the muscle volume difference between the infraspinatus and teres minor (Table 5), significant correlations were observed with sex, symptom duration, work and sports activity level, Hill-Sachs lesion, and preoperative range of motion for Dif.pre.1mo in the Y section.

With regard to the CT density difference of the subscapularis muscle (Table 6), significant correlations were observed only with the side and sulcus signs for Dif.pre.1mo in the Y+5 cm section.

With regard to the CT density difference between the infraspinatus and teres minor muscles (Table 7), a significant correlation was observed only with the sulcus sign for Dif.pre.1mo in the Y and Y+5 cm sections.

## Discussion

In this study, we evaluated serial changes in muscle volume and CT density in each shoulder muscle of patients with ASI during the perioperative period of Bankart repair surgery using 3D modeling-based sectional measurements. The findings suggested that overall muscle volume and CT density decreased at 1 month postoperatively compared with the preoperative values and recovered by 12 months postoperatively. The mean VR_Ssc/Isp+TM_ decreased in all sections at 1 month postoperatively compared with the preoperative values but increased afterward, whereas the mean CT-DR_Ssc/Isp+TM_ in all sections remained mostly unchanged throughout the perioperative period. Moreover, perioperative differences in muscle volume may be correlated with sex, work and sports activity level, Hill-Sachs lesion, and range of motion. The perioperative differences in CT density were not correlated with most factors, except for sulcus signs.

In recent years, muscle atrophy and fatty degeneration have frequently been discussed in relation to osteoarticular diseases. Several studies have investigated the recovery of muscle volume after rotator cuff repair; however, few reports exist on other shoulder disorders. Of note, one study evaluated shoulder muscle cross-sectional areas in cases of shoulder joint instability [14]. Ishikawa et al. [14] found that the muscle area of Isp+TM was smaller than that of Ssc in anterior instability, whereas the muscle area of Ssc was smaller than that of Isp+TM in posterior and multidirectional instabilities. Our group also investigated this question using a sectional volume measurement approach. The results suggested that the volume ratio of the shoulder muscles (i.e., VR_Ssc/Isp+TM_) was higher in shoulders with ASI than in nonpathological shoulders [15]. Assessing the muscular imbalance of the transverse force couple in shoulder instability may be valuable because it may relate to the muscular force balance. However, previous studies have examined the preoperative status of shoulder muscle imbalance, but none have examined changes in muscle imbalance pre- and postoperatively. Therefore, this study appears to be the first to examine muscle changes associated with shoulder instability during the perioperative period.

The present study demonstrated that late postoperative volume changes in the shoulder muscles were negatively correlated with those in the early postoperative period. Notably, the same tendency was observed in a previous study [17] by our group on serial volume changes in the shoulder muscles after rotator cuff repair. Xu et al. [18] also examined changes in the supraspinatus muscle volume after rotator cuff surgery and found that it decreased transiently during the first 3 months but recovered to baseline by 12 months postoperatively. However, the reversibility of muscle atrophy after surgical repair remains controversial and inconclusive, as changes in muscle volume after surgery vary from increases to decreases [10,11]. Additionally, a fundamental difference between rotator cuff repair and shoulder instability is that the latter does not involve repairing a torn tendon; after rotator cuff repair, an increase in musculotendon volume is expected, whereas in shoulder instability, no increase in volume is anticipated. However, both groups showed similar trends in muscle volume changes; therefore, these muscle changes may reflect the natural course of the surgical shoulder, regardless of the type of surgical treatment.

This study also revealed that postoperative changes in CT density of the shoulder muscles showed a similar tendency to changes in muscle volume. Muscle CT density and volume are indicators of muscle atrophy and degeneration [19]. In the shoulder joint, a strong correlation has been observed between the CT density of rotator cuff muscle volume and the visual assessment of fatty degeneration in patients with rheumatoid arthritis [20]. One possible hypothesis is that muscle CT density decreases in parallel with muscle atrophy and fatty degeneration in the early postoperative period and recovers in the late postoperative period. However, the present results cannot be explained solely by muscle atrophy and fatty degeneration, as CT density may also reflect other factors such as edematous changes.

The balance of antagonistic muscle forces can be evaluated using the volume ratio and CT density ratio of the shoulder muscles in the transverse force couple. Our previous study demonstrated that the volume ratio (VR_Ssc/Isp+TM_) and the CT density ratio (CT-DR_Ssc/Isp+TM_) were nearly balanced in nonpathological shoulders. In patients with ASI, the VR_Ssc/Isp+TM_ was relatively high, whereas the CT-DR_Ssc/Isp+TM_ was relatively low, implying a possible imbalance between the anterior and posterior shoulder muscles [15]. The present results demonstrated that VR_Ssc/Isp+TM_ changed as the other parameters changed, but CT-DR_Ssc/Isp+TM_ remained mostly unchanged throughout the perioperative period. This finding implied that the difference in muscle CT density was too small to be statistically significant compared with the difference in muscle volume. Therefore, discrepancies may exist between the two ratios, making direct comparison difficult.

These discrepancies arose in the present study when we considered changes in muscle volume and CT density during the perioperative period, each of which appeared to have different clinical implications. Regarding muscle volume, perioperative differences were correlated with sex, work and sports activity level, and Hill-Sachs lesions. Since Bankart repair stabilizes the bony and labral structures, the muscle-tendon system is preserved. Thus, recovery from perioperative wasting and atrophy may occur primarily through mechanisms unrelated to muscle-tendon injury rather than changes directly associated with the injury itself. In our previous study [17] on muscle volume changes after rotator cuff repair, preoperative history and life activity levels were correlated with postoperative muscle volume differences in non-tear site muscles. Work and sports activity levels and sex were naturally well correlated with postoperative muscle volume changes in our results because such parameters may influence muscle recovery capacity. Additionally, the Hill-Sachs lesion, a bony defect in the humeral head caused by compression associated with ASI, becomes larger when the compression force is greater and exerted for a longer duration (i.e., chronic subluxation) [21,22]. Symptom duration was also correlated with postoperative muscle volume changes in the present study. In chronic muscle-tendon lesions, recovery from muscle volume loss is difficult and prolonged because chronic lesions can cause irreversible structural changes, and increased muscle stiffness and tension reduce repair and healing potential [23].

By contrast, with regard to CT density, perioperative differences were not correlated with most factors, except for sulcus signs. The sulcus sign assesses inferior shoulder capsule laxity [24]. However, the relationship between differences in muscle CT density and sulcus signs lacks a clear explanation. Notably, no correlation was observed with other assessments of laxity, such as the load and shift tests, or differences in muscle CT density. While CT density measurement may be a viable method for monitoring muscle degeneration, it can produce discrepant results in this situation, possibly due to small statistical differences. Additionally, studies utilizing CT density are limited because of the inevitable issue of radiation exposure. By contrast, quantitative assessments of muscle degeneration, such as the fat fraction ratio, have been more widely adopted as safe and reliable methods.

This study has several limitations. First, the sample size was small. Not all patients who underwent Bankart repair surgery were included because we limited our study to those with consecutive CT scans. Additionally, as a single-center study, generalizability is limited. Furthermore, we could not evaluate the entire muscle in ASI cases; the sectional measurement approach adopted may have underestimated relative volume changes. These issues need to be addressed in future studies.

## Conclusions

Our study demonstrated that shoulder muscle volume and CT density in patients with ASI decreased during the early postoperative period of Bankart repair surgery compared with the preoperative period and recovered during the late postoperative period. The mean VR_Ssc/Isp+TM_ decreased in all sections in the early postoperative period but increased afterward, whereas the mean CT-DR_Ssc/Isp+TM_ in all sections remained mostly unchanged throughout the perioperative period. Furthermore, perioperative differences in muscle volume may be correlated with sex, work and sports activity level, Hill-Sachs lesion, and range of motion. Perioperative differences in CT density were not correlated with most factors, except for sulcus signs. Changes in muscle volume and CT density during the perioperative period may provide meaningful information regarding muscle balance and recovery. However, further studies are needed to assess their clinical validity.
